# Perinatal Parenting Stress, Anxiety, and Depression Outcomes in First-Time Mothers and Fathers: A 3- to 6-Months Postpartum Follow-Up Study

**DOI:** 10.3389/fpsyg.2016.00938

**Published:** 2016-06-24

**Authors:** Laura Vismara, Luca Rollè, Francesca Agostini, Cristina Sechi, Valentina Fenaroli, Sara Molgora, Erica Neri, Laura E. Prino, Flaminia Odorisio, Annamaria Trovato, Concetta Polizzi, Piera Brustia, Loredana Lucarelli, Fiorella Monti, Emanuela Saita, Renata Tambelli

**Affiliations:** ^1^Department of Pedagogy, Psychology, Philosophy, University of CagliariCagliari, Italy; ^2^Department of Psychology, University of TorinoTorino, Italy; ^3^Faculty of Psychology, University of BolognaBologna, Italy; ^4^Department of Psychology, University Cattolica del Sacro CuoreMilano, Italy; ^5^Department of Dynamic and Clinical Psychology, Sapienza University of RomaRoma, Italy; ^6^Department of Psychological, Educational and Training Sciences, University of PalermoPalermo, Italy

**Keywords:** transition to parenthood, mothers, fathers, parenting stress, perinatal anxiety, postnatal depression, follow-up study

## Abstract

**Objective:** Although there is an established link between parenting stress, postnatal depression, and anxiety, no study has yet investigated this link in first-time parental couples. The specific aims of this study were 1) to investigate whether there were any differences between first-time fathers’ and mothers’ postnatal parenting stress, anxiety, and depression symptoms and to see their evolution between three and 6 months after their child’s birth; and 2) to explore how each parent’s parenting stress and anxiety levels and the anxiety levels and depressive symptoms of their partners contributed to parental postnatal depression.

**Method:** The sample included 362 parents (181 couples; mothers’ *M*_Age_ = 35.03, *SD* = 4.7; fathers’ *M*_Age_ = 37.9, *SD* = 5.6) of healthy babies. At three (T1) and 6 months (T2) postpartum, both parents filled out, in a counterbalanced order, the Parenting Stress Index-Short Form, the Edinburgh Postnatal Depression Scale, and the State-Trait Anxiety Inventory.

**Results:** The analyses showed that compared to fathers, mothers reported higher scores on postpartum anxiety, depression, and parenting stress. The scores for all measures for both mothers and fathers decreased from T1 to T2. However, a path analysis suggested that the persistence of both maternal and paternal postnatal depression was directly influenced by the parent’s own levels of anxiety and parenting stress and by the presence of depression in his/her partner.

**Discussion:** This study highlights the relevant impact and effects of both maternal and paternal stress, anxiety, and depression symptoms during the transition to parenthood. Therefore, to provide efficacious, targeted, early interventions, perinatal screening should be directed at both parents.

## Introduction

In both men and women, the transition to parenthood involves physical, hormonal, neurochemical, and neurobiological shifts (Wisner et al., 2006; Feldman, 2007; Slade et al., 2009; Kim et al., 2010); psychological changes concerning identity, affect, representations, and cognition (Stern, 1995; Ammaniti et al., 2014); and socio-relational adjustments (Cowan and Cowan, 1995, 2000; Bost et al., 2002). The involved personal and family changes may lead to increased vulnerability to psychological distress (Epifanio et al., 2015). In this context, postnatal depression (PND) is a major parental mental health issue (Miller, 2002; Rollè et al., 2011; Parfitt and Ayers, 2014). PND occurs in about 15–20% of mothers in Western countries and may have severe consequences on maternal and family wellbeing, along with affecting child development (O’Hara and Wisner, 2014; Tambelli et al., 2014a).

In the past two decades, several studies have shown that fathers also experience postpartum symptomatology, particularly perinatal depression (Condon et al., 2004; Paulson and Bazemore, 2010); a recent study found the prevalence of paternal perinatal depression to be 10.4% between the beginning of first trimester of pregnancy and the end of the first postpartum year, with an increase to 25% between three and 6 months after birth (Paulson and Bazemore, 2010). Paternal perinatal depression can lead to inadequate parental functioning (Wilson and Durbin, 2010) and negative child outcomes (Ramchandani et al., 2005, 2008; Tambelli et al., 2014b). In addition, fathers seem to follow their partners’ mood and emotional states, increasing the possibility of negative outcomes for children (Deater-Deckard, 1998; Nishimura and Ohashi, 2010).

This body of evidence thus highlights the need for research focused on identifying the factors that play a role in the onset and chronicity of PND in both mothers and fathers. Anxiety disorder has been shown to increase the risk of developing or triggering depression (Iliadis et al., 2015). In particular, perinatal anxiety, a frequent psychopathological condition in mothers (Teixeira et al., 2009; O’Hara and Wisner, 2014; Martini et al., 2015), has been identified as a relevant risk factor for perinatal depression (Norhayati et al., 2015). Worries, preoccupations, generalized anxiety, and/or specific phobias (e.g., tocophobia) can persist during pregnancy and in the postnatal period (Fenaroli and Saita, 2013). Perinatal anxiety in men has been analyzed in a limited number of studies; however, throughout the perinatal period, compared to mothers, fathers appear to have lower levels of anxiety (Matthey et al., 2000, 2003; Figueiredo and Conde, 2011; Candelori et al., 2015).

A consistent association between PND and parenting stress has also been identified by several studies (Yim et al., 2015). Anding et al. (2016) found that perceived parental stress was the strongest predictor of depressive symptoms in both mothers and fathers at 2 weeks postpartum; perceived stress in fathers at 6 weeks postpartum was found to be a predictor of paternal postpartum depression at 12 weeks in a study by Kamalifard et al. (2014). Parenting stress may involve how fathers and mothers experience their parental role, parental perceptions of how difficult the infant is, and the quality of parent–child interactions. Stress associated with the parental domain has shown to be linked to depressive symptoms, whereas findings regarding child and parent–child interactions are inconsistent (Thomason et al., 2014). The clinical relevance of parenting stress with regard to its implications on parental behavior and child outcomes make it a crucial variable requiring deeper investigation.

To our knowledge, only a few studies have examined the relationship between PND, anxiety, and parenting stress in the first few months after birth, and if and how these variables may contribute to higher levels of PND in mothers and fathers. In a correlational longitudinal study on maternal stress, depressive symptoms, and anxiety, Liou et al. (2014) found a low to high degree of correlation in maternal stress, depressive symptoms and anxiety in pregnancy and postpartum. In addition, the three types of maternal distress had different courses: levels of depressive symptoms remained unchanged; anxiety levels increased as gestation advanced but declined after birth, and stress decreased gradually during pregnancy but increased after birth. In fathers, Wee et al. (2015) explored the relationship among the three variables during pregnancy and found that high levels of anxiety early in pregnancy predicted high levels of depression and stress in late pregnancy. At 2- to 3-months postpartum, Goodman (2008) showed that maternal PND was associated with increased paternal depression and higher paternal parenting stress and that depressed women’s partners had less optimal interactions with their infants, indicating that fathers do not compensate for the negative effects of maternal depression on the child. We also know that in the offspring of depressed parents, a second parent with emotional problems is likely to increase the risk of emotional disorders (Landman-Peeters et al., 2008); thus, it is important to include partners’ mental health when exploring parental PND and to consider the couple as a whole.

These links suggest the need for a better understanding of the reciprocal influences among mothers, fathers, and their infants in the perinatal period. A longitudinal approach may provide further information on the relationships between anxiety, parenting stress, and PND in the postpartum period. Based on the above empirical and clinical evidence, the present longitudinal study had the following aims:

• To examine whether there are any differences and relationships between fathers’ and mothers’ levels of parenting stress, anxiety, and depression symptoms and to evaluate their evolution from three to 6 months after their child’s birth.
• To explore, through a path model, whether the persistence of PND could be a response to the parent’s own parenting stress and anxiety levels and the anxiety levels and depressive symptoms of his/her partner.

This study was part of a larger, ongoing longitudinal study on maternal and paternal depression in first-time parents and the development of their children’s affective regulation. In this paper, we present data concerning parents who completed the first (Time 1) and second step (Time 2) of the assessment at the third and sixth month after the child’s birth.

## Material and Methods

### Participants

The study participants were 362 parents (181 couples) and their healthy 208 babies (55.8% boys, 44.2% girls). Of these, 70% were married couples and 30% were cohabiting; 6% of the mothers and 12% of the fathers had an elementary school qualification, 34% of the mothers and 45% of the fathers had a high-school qualification, 47% of the mothers and 38% of the fathers a college degree, and 13% of the mothers and 5% of the fathers had a PhD. Mothers’ mean age ranged from 20 to 49 years (*M*_Age_ = 35.03 years, *SD* = 4.7 years), and fathers’ mean age ranged from 20 to 54 years (*M*_Age_ = 37.9 years, *SD* = 5.6 years). The median income of the parents belonged to the Italian middle working class and socio-economic status as assessed by a detailed questionnaire and according to ISTAT classification (Istituto Nazionale di Statistica [ISTAT], 2013). No participant was undergoing medical/psychological treatment at the time of assessment.

### Measures

The Edinburgh Postnatal Depression Scale (EPDS; Cox et al., 1987) is a self-report questionnaire including 10-items addressing depression symptoms occurring within the previous seven days. The total score is calculated by adding the individual items on a 4-point likert scale. There were two adopted cut-off scores: 8/9, as suggested in the EPDS Italian validation (Benvenuti et al., 1999), and 12/13, as suggested by Cox et al. (1987) to identify more severe depression. In the current study, the internal consistency coefficient for the mothers was α = 0.84 at 3 months and α = 0.81 at 6 months; for the fathers, it was α = 0.81 at 3 months and α = 0.78 at 6 months.

The State-Trait Anxiety Inventory (STAI; Spielberger et al., 1983; Pedrabissi and Santinello, 1989) is a commonly used self-report measure of trait and state anxiety. STAI has 20 items for assessing trait anxiety (STAI-T) and 20 for state anxiety (STAI-S). All items are rated on a 4-point scale (i.e., from “Almost Never” to “Almost Always”). The adopted cut-off score was > 40, as suggested by the Italian validated version (Pedrabissi and Santinello, 1989). In the current study, the internal consistency coefficient for the mothers was α = 0.95 at 3 months and α = 0.94 at 6 months; for the fathers, it was α = 0.95 at 3 months and α = 0.94 at 6 months.

The Parenting Stress Index—Short Form (PSI-SF; Abidin, 1995; Guarino et al., 2008) is a self-report instrument that measures stress specifically associated with parenting. The PSI-SF consists of 36 statements referring to the past week. All items are rated on a 5-point scale. Parents who obtain a total stress score above the 90th percentile or a raw score of 90 are considered to experience clinically significant parenting stress, as indicated by the Italian validation (Guarino et al., 2008). The total stress score is a composite score of the subscale scores: parental distress, parent–child dysfunctional interaction, and difficult child. In the current study, the internal consistency coefficient for the mothers was α = 0.94 at 3 months and α = 0.92 at 6 months; for the fathers, it was α = 0.92 at 3 months and α = 0.94 at 6 months.

### Procedure

The research project obtained approval from the hospital and university ethics committees. All participants signed a written informed consent form.

Time 1 data were collected approximately 3 months after birth, while Time 2 data were collected approximately 6 months after birth. Parents who met selection criteria and agreed to participate independently completed at home a demographics questionnaire, the EPDS, STAI, and PSI-SF at both Time 1 and Time 2.

### Data Analysis

Data analysis was conducted with IBM SPSS Version 21 and IBM SPSS Amos 21. Since the mother and father in each couple were considered as dependent, all comparisons between mothers and fathers used statistical methods for paired data. Descriptive statistics were calculated on the assessed psychological variables, reporting frequencies, percentages, mean values, and standard deviation.

To analyze changes over time and to analyze the differences between mothers and fathers in anxiety, depression, and parenting stress scores, we used a paired sample *t*-test, marginal homogeneity (Agresti, 1990), and McNemar’s exact test. Pearson’s correlations were used to assess the association between maternal and paternal scores and Time 1 and Time 2 scores.

An exploratory model of maternal and paternal PND was tested by path analysis. Specifically, by taking into account the proven impact of parenting stress and partner support on the onset of PND, we tested whether parenting stress and anxiety and the anxiety levels and depressive symptoms of partners had direct effects on PND.

Evaluation of model fit was based on a χ^2^ test, with a statistical significance level of less than 0.05 indicating inadequate fit (Allison, 2003), along with the recommended minimal set of fit indices, including the Tucker–Lewis Index (TLI ≥ 0.95) the comparative fit index (CFI ≥ 0.95), the root mean square error of approximation (RMSEA ≤ 0.06), and the standardized root mean square residual (SRMR *<* 0.1; Allison, 2003; Tabachnick and Fidell, 2007).

## Results

### Comparisons between Mothers and Fathers

Frequency, mean values, and standard deviation were calculated for each considered variable.

The results from the non-parametric tests (**Table 1**) indicate that the mothers were more likely to experience depression and anxiety than their partners. Specifically, the marginal homogeneity test for EPDS, which analyzed data regarding normal functioning (EPDS < 9), borderline (EPDS total 9–12), and depressed (EPDS total ≥13) parents, showed a difference between the mothers and fathers within each couple, showing that the mothers were more depressed than their partners. McNemar’s exact test for STAI, which analyzed data regarding normal functioning (STAI < 40) and anxious (STAI > 40) parents, revealed that the mothers were more anxious than their partners. Similarly, a paired sample *t*-test showed differences between EPDS and STAI mean scores for the mothers and fathers in each couple (**Table 1**), with women showing higher scores than men.

**Table 1:** Distribution of outcomes by mother and father couples.

	Time 1 (3 Months)				Time 2 (6 Months)			
	Mothers	Fathers	*t*	*p*-value	Mothers	Fathers	*t*	*p*-value
**EPDS**
Mean score (*SD*)	7.8 (5.6)	4.8 (4.5)	9.04	<0.001^a^	5.9 (4.3)	4.3 (3.6)	5.4	<0.001^a^
Normal (<9) *N (%)*	114 (63%)	152 (84%)			143 (79%)	156 (86.2%)		
Borderline (9–12) *N* (%)	36 (19.9%)	17 (9.4%)		<0.001	21 (11.6%)	21 (11.6%)		
Depressed (>13) *N* (%)	31 (17.1%)	12 (6.6%)			17 (9.4%)	4 (2.2%)		0.002^b^
**STAI**
STAI – State
Mean score (*SD*)	38.8 (11.1)	34.9 (9.4)	6.59	0.000^a^	35.2 (9.8)	33.6 (9.3)	2.65	0.01^a^
Normal (<40) *N* (%)	111 (61.3%)	135 (74.6%)		<0.001^c^	131 (72.4%)	139 (76.8%)		0.256^c^
Anxious (>40) *N* (%)	70 (38.7%)	46 (25.4%)			50 (27.6%)	42 (23.2%)		
STAI – Trait
Mean score (*SD*)	39.2 (11)	35.1 (9.4)	6.07	<0.001^a^	37 (9)	32.9 (8.4)	6.80	<0.001^a^
Normal (<40) *N* (%)	102 (56.4%)	134 (74%)		<0.001^c^	117 (64.6%)	145 (80.1%)		<0.001^c^
Anxious (>40) *N* (%)	79 (43.6%)	47 (26%)			64 (35.4%)	36 (19.9%)		
**PSI**
PD Mean score (*SD*)	24.3 (7.9)	22.1 (8)	4.73	<0.001^a^	22.41 (7.4)	21.6 (7.3)	0.48	0.629^a^
PD Mean score (*SD*)	19 (7.3)	19.3 (7.4)	-0.77	0.443^a^	18.2 (5.9)	18.1 (6)	0.34	0.732^a^
DC Mean score (*SD*)	21.7 (7.5)	22.4 (7)	-2.17	0.031^a^	21.3 (7.2)	20.9 (7)	0.93	0.353^a^
**Total stress**
Mean score (*SD*)	65 (20.3)	63.8 (20.5)	1.10	0.271^a^	61.9 (17.1)	60.6 (17.8)	1.27	0.205^b^
Normal (<90) *N* (%)	177 (85.1%)	175 (84.1%)		0.791^c^	200 (96.2%)	195 (93.8%)		0.227^c^
Clinical stress (>90) *N* (%)	31 (14.9%)	33 (15.9%)			8 (3.8%)	13 (6.3%)		

*EPDS, Edinburgh Postnatal Depression Scale; STAI, State and Trait Anxiety Inventory; PSI, Parenting Stress Index—Short Form; PD, Parental Distress; P-CDI, Parent–Child Dysfunctional Interaction; DC, Difficult Child. ^a^Paired t-test, ^b^marginal homogeneity test, and ^c^McNemar’s exact test.*

As concerns PSI, McNemar’s exact test for total stress, which analyzed data regarding normal functioning (total stress <90) and clinically stressed (total stress >90) parents, did not reveal any statistically differences between the mothers and fathers in each couple. Instead, differences between the mothers and fathers within each couple were found at Time 1 with respect to the parental distress and difficult child subscales, showing that compared to their partners, the mothers reported higher levels of psychological distress and perceived their children as being more difficult.

### Time 1 versus Time 2 Evaluations

Because the psychological variables differed between the mothers and fathers, we investigated the main effect of time on each parent separately. Results are reported in **Table 1**. Significant differences emerged between Time 1 and Time 2 regarding specific psychological variables, mostly within the mothers. In particular, the marginal homogeneity test showed that the number of depressed mothers decreased from Time 1 to Time 2 (*p* < 0.001). Similarly, McNemar’s exact test showed that the mothers’ state anxiety decreased from Time 1 to Time 2 (*p* = 0.014).

Among the mothers, the mean EPDS, STAI-S, and STAI-T scores decreased from Time 1 to Time 2: *t* (180) = 5.14 and *p* < 0.001, *t* (180) = 3.98 and *p* < 0.001, and *t* (180) = 2.65 and *p* = 0.009, respectively. With respect to maternal PSI, McNemar’s exact test showed that the mothers’ clinical stress decreased from Time 1 to 3.8% Time 2 (*p* < 0.001). Both mean total stress and parental distress (PD) subscale scores decreased from Time 1 to Time 2: *t* (207) = 2.62 and *p* < 0.001, and *t* (207) = 4.21 and *p* < 0.001, respectively.

Among the fathers, EPDS, STAI-S, and STAI-T categorical scores did not show any significant differences across time. Regarding mean scores, only the mean STAI-T score decreased from Time 1 to Time 2: *t* (180) = 3.21 and *p* = 0.002. With respect to paternal PSI, McNemar’s exact test showed that the fathers’ clinical stress decreased from Time 1 to Time 2 (*p* = 0.001). The mean total stress and parent–child dysfunctional interaction (P-CDI) subscale scores decreased from Time 1 to Time 2: *t* (207) = 2.53 and *p* = 0.012, and *t* (207) = 2.47 and *p* = 0.014, respectively.

### Association between Parenting Stress, Anxiety, and Depression

The Pearson correlation coefficients between the EPDS, STAI-S, STAI-T, and PSI scores during the third month after the child’s birth were positively correlated with the EPDS, STAI-S, STAI-T, and PSI scores at 6 months postpartum.

### Association between Mothers’ and Fathers’ Parenting Stress, Anxiety, and Depression Scores

The Pearson correlation coefficients between the maternal and paternal scores are reported in **Table 2**. The EPDS, STAI-S, STAI-T, and PSI maternal scores were strongly positively correlated with the EPDS, STAI-S, STAI-T, and PSI paternal scores at both Time 1 and Time 2.

**Table 2:** Bivariate correlation between mothers’ and fathers’ parenting stress, anxiety, and depression scores at Time 1 and Time 2

Mothers								
Fathers	EPDS 3	STAI-S 3	STAI-T 3	STRESS 3	EPDS 6	STAI-S 6	STAI-T 6	STRESS 6
EPDS 3	0.54^∗∗^	0.33^∗∗^	0.28^∗∗^	0.33^∗∗^	0.23^∗∗^	0.28^∗∗^	0.26^∗∗^	0.22^∗∗^
STAI-S 3	0.40^∗∗^	0.47^∗∗^	0.38^∗∗^	0.52^∗∗^	0.21^∗∗^	0.30^∗∗^	0.28^∗∗^	0.29^∗∗^
STAI-T 3	0.36^∗∗^	0.41^∗∗^	0.38^∗∗^	0.49^∗∗^	0.19^∗∗^	0.24^∗∗^	0.22^∗∗^	0.19^∗∗^
STRESS 3	0.38^∗∗^	0.33^∗∗^	0.29^∗∗^	0.74^∗∗^	0.30^∗∗^	0.34^∗∗^	0.34^∗∗^	0.51^∗∗^
EPDS 6	0.27^∗∗^	0.07	0.08	0.21^∗∗^	0.44^∗∗^	0.39^∗∗^	0.32^∗∗^	0.34^∗∗^
STAI-S 6	0.29^∗∗^	0.18^∗∗^	0.18^∗∗^	0.25^∗∗^	0.40^∗∗^	0.53^∗∗^	0.50^∗∗^	0.41^∗∗^
STAI-T 6	0.32^∗∗^	0.11^∗^	0.11^∗^	0.29^∗∗^	0.44^∗∗^	0.50^∗∗^	0.49^∗∗^	0.46^∗∗^
STRESS 6	0.21^∗∗^	0.12^∗^	0.10	0.35^∗∗^	0.35^∗∗^	0.45^∗∗^	0.48^∗∗^	0.60^∗∗^

*EPDS3, Edinburgh Postnatal Depression scores at 3 months after the child’s birth; STAI-S 3, State Anxiety Inventory scores at 3 months after the child’s birth; STAI-T 3, Trait Anxiety Inventory scores at 3 months after the child’s birth; STRESS 3, Parenting Stress Index Total Stress scores at 3 months after the child’s birth; EPDS 6, Edinburgh Postnatal Depression scores at 6 months after the child’s birth; STAI-S 6, State Anxiety Inventory scores at 6 months after the child’s birth; STAI-T 6, Trait Anxiety Inventory scores at 6 months after the child’s birth; STRESS 3, Parenting Stress Index Total Stress scores at 6 months after the child’s birth. ^∗^*p* < -0.05; ^∗∗^*p* < 0.01*

### Path Models

The next step was to analyze two hypothesized path models for mothers and fathers, respectively. The basic strategy involved constructing two separate conceptual models based on theoretical evidence that a parent’s own parenting stress and anxiety levels (at Time 1 and Time 2) and the anxiety levels and depressive symptoms of his/her partner (at Time 1 and Time 2) precede postpartum depression (at Time 1 and Time 2). The two models contained four exogenous variables (own parenting stress and anxiety levels and partner’s anxiety and depressive symptoms at Time 1), which were assumed to be correlated.

The conceptual model for mothers yielded a poor fit: χ^2^= 181,818 *df* = 18 *p* < 0.001, CFI = 0.810, TLI = 0.619, RMSEA = 0.210, and SRMR = 0.123. To develop a parsimonious model, we deleted any non-significant statistically paths (the weakest paths were deleted first) until all paths were significant (MacCallum, 1986). Deleting the non-significant statistically paths resulted in a significantly improved model**:** χ^2^= 6.215, *df* = 6 *p* = 0.400, CFI = 0.973, TLI = 0.999, RMSEA = 0.01, and SRMR = 0.023.

**FIGURE 1:**
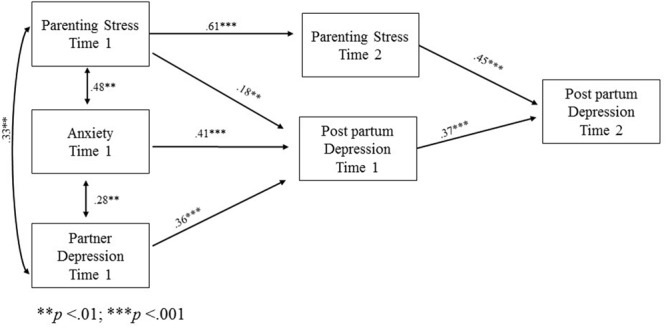
**Path model with statistically significant beta standardized coefficients for mothers**.

The final model (**Figure 1**) for mothers found Parenting Stress at Time 1, Anxiety at Time 1, and Partner Depression at Time 1 all serving as exogenous variables that were correlated with each other. Both own parenting stress and trait anxiety levels and the depressive symptoms of the partner at Time 1 had a direct effect on own postpartum depression at Time 1 and indirect effect on own postpartum depression at Time 2. Own parenting stress at Time 1 had a direct effect on parenting stress at Time 2. Finally, in turn, Parenting Stress at Time 2 and postpartum depression at Time 1 had a direct effect on own postpartum depression at Time 2. There was no direct relationship between postpartum depression at Time 1 and own parenting stress at Time 2.

Similarly, the conceptual model for fathers yielded a poor fit: χ^2^= 247.729 *df* = 26 *p* < 0.001, CFI = 0.761, TLI = 0.586, RMSEA = 0.203, and SRMR = 0.162. Any non-significant statistically pathways were removed one after another, with the least significant pathway being removed at each step to refine the model. This process continued until all paths were significant. Deleting the non-significant statistically paths resulted in considerably improved fit statistics: χ^2^= 5.121, *df* = 6, *p* = 0.528, CFI = 0.999, TLI = 0.999, RMSEA = 0.000, and SRMR = 0.021.

**FIGURE 2:**
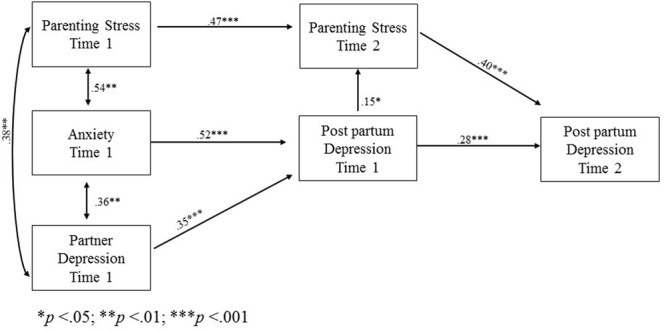
**Path model with statistically significant beta standardized coefficients for fathers**.

The final model for fathers (**Figure 2**) found Parenting Stress at Time 1, Anxiety at Time 1, and Partner Depression at Time 1 all serving as exogenous variables that were correlated with each other.

Both own trait anxiety levels and the depressive symptoms of the partner at Time 1 had a direct effect on own postpartum depression at Time 1 and indirect effect on own postpartum depression at Time 2. Both own trait anxiety levels and the depressive symptoms of the partner at Time 1 had an indirect effect on Parenting Stress at Time 2.

Finally, own parenting stress at Time 1 had a direct effect on parenting stress at Time 2 and indirect effect on postpartum depression at Time 2.

**Table 3** show the estimates of indirect effects and 95*%* bias*-*corrected confidence interval (CI) for mothers and fathers models. All indirect pathways were significant in each model.

**Table 3:** Specific indirect effects and confidence intervals of the path models for mothers and fathers.

	Indirect effect	Bias-corrected 95% CI
**Path for mothers**
PS T1 → PS T2 → PPD T2	0.28^∗∗^	0.21–0.36
PS T1 → PPD T1→ PPD T2	0.07^∗∗^	0.02–0.14
ANX T1→ PPD T1→ PPD T2	0.15^∗∗^	0.09–0.22
PD T1→ PPD T1→ PPD T2	0.14^∗∗^	0.07–0.21
**Path for fathers**
PS T1 → PS T2 → PPD T2	0.19^∗∗^	0.10–0.29
ANX T1→ PPD T1→ PPD T2	0.15^∗∗^	0.06–0.23
PD T1→ PPD T1→ PPD T2	0.10^∗∗^	0.04–0.17
ANX T1→ PPD T1→ PS T2→PPD T2	0.04^∗^	0.01–0.07
PD T1→ PPD T1→ PS T2→ PPD T2	0.03^∗^	0.00–0.06
ANX T1→ PPD T1→ PS T2	0.08^∗^	0.02–0.13
PD T1→ PPD T1→ PS T2	0.05^∗^	0.01–0.11
PD T1→ PPD T1→ PPD T2	0.12^∗∗^	0.06–0.19

*Table values are standardized coefficients. T1, Time 1; T2, Time 2; PS, Parenting stress; PPD, Postpartum depression; ANX, Anxiety; PD, Partner depression.*

*^∗^Significant indirect effect for *p* < 0.05. ^∗∗^Significant indirect effect for *p* < 0.01.*

Our results thus suggest that both maternal and paternal postpartum depression were influenced directly and indirectly by a parent’s own levels of anxiety and parenting stress as well as by the presence of depression in his/her partner. Although the two models are similar, they differ with respect to the role of parenting stress. The latter was shown to have an effect on maternal postpartum depression at 3 months postpartum, whereas it only influences paternal postpartum depression 6 months after the child’s birth.

## Discussion

This study provides an analysis of men’s and women’s emotional experiences connected to the transition to parenthood and their mental health in the first few months after the birth of their first child. In particular, our study had two main aims: to investigate the differences and relationships between fathers’ and mothers’ parenting stress, anxiety, and depression symptoms and to explore, through a path model, whether the persistence of postpartum depression could be linked to a parent’s own parenting stress and anxiety levels and his/her partner’s anxiety levels and depressive symptoms.

The predominant focus of postnatal research in both women and men has been on depression (Brustia et al., 2009; Yelland et al., 2010). Nevertheless, there is growing evidence that anxiety is also present among first-time mothers (Reck et al., 2008; Rowe et al., 2008; Fisher et al., 2010; Matthey et al., 2013), and some data suggest the same is true for men (Matthey et al., 2000; Matthey et al., 2003). Parenting stress is also associated to PND (Gelfand et al., 1992; Cornish et al., 2006; Sidor et al., 2011). The strength of this study is its exploration of parenting stress, depressive and anxiety symptoms in first-time parental couples and their causal relationship across time.

With respect to the study’s first aim, compared to the fathers, the mothers in our research had higher scores of depression and anxiety at three and 6 months postpartum. These results confirm the data from the literature, highlighting how women, compared to men, seem more vulnerable to emotional difficulties throughout the perinatal period (Matthey et al., 2000; Matthey et al., 2003; Figueiredo and Conde, 2011; O’Hara and Wisner, 2014). The mothers showed positive changes between three and 6 months postpartum, with decreased depression and anxiety, whereas in this same period, only the trait anxiety scores decreased in fathers. Both perinatal depressive and anxiety symptoms tend to decrease after birth; therefore, our results seem to confirm the findings of other studies (Figueiredo and Conde, 2011; Agrati et al., 2015).

Moreover, when looking at the PSI scores across time, the total stress score decreased for both parents between three and 6 months after birth. Differences emerged with respect to the subscales; specifically, the mothers showed a decrease in the parental distress subscale, whereas fathers showed a decrease in the parent–child dysfunctional interaction subscale. Such findings are in line with those of Seah and Morawska (2016), who analyzed the levels of parenting distress in both parents within the first 6 months of life. Such results may be due to the fact that, compared to fathers, mothers are more involved in caring for the baby straight away; this may represent a specific stressor that distinguishes mothers from fathers in the aftermath of birth.

Our outcomes also showed that for both parents, the scores at 3 months postpartum were correlated to those at 6 months, which may suggest how maternal and paternal emotional experiences are connected and influence each other, as previously highlighted by several studies (Matthey et al., 2000, 2003; Baldoni et al., 2009; Paulson and Bazemore, 2010). These results may have important clinical implications for optimal PND prevention and care programs. Thus, accurate assessments of depression in both parents, not just mothers, should be developed and implemented to take into account the possible reciprocal influence on mood and symptomatology.

To summarize, in our research, and in line with previous studies (Kim and Swain, 2007; Paulson and Bazemore, 2010; Figueiredo and Conde, 2011; Don and Mickelson, 2012), compared to fathers, mothers have higher scores on all self-report measures of parenting stress, anxiety, and depression, and all measures decrease from the third to the sixth month after childbirth. In addition, the focus on the mothers and fathers of the same child, as measured through statistical methods for paired data, highlighted that rather than considering mothers and fathers independently, it is important to acknowledge the interplay of partners’ psychological status within a parental couple in order to provide successful interventions.

With respect to the study’s second aim, the findings indicate that the onset of depressive symptoms in both mothers and in fathers was influenced by their own levels of anxiety and parenting stress as well as by the presence of depression in their partners. With respect to anxiety, the literature shows that it is a relevant risk factor for PND in both first-time mothers (Robertson et al., 2004; Grant et al., 2008; Coelho et al., 2011) and first-time fathers (Ferketich and Mercer, 1995; Robertson et al., 2004; Wee et al., 2011). Our results are in line with previous studies finding that in the postpartum period, high levels of anxiety and stress are the strongest predictors of elevated depressive symptoms in men. Anxiety might also challenge parents’ ability to initiate and maintain positive affective interactions with their children and partners. For this reason, our findings highlight the need to screen both mothers’ and father’ psychological status.

With regard to parenting stress, we embraced Abidin’s (1995) definition, which states that parenting stress is the gap between the demands associated with the parenting role and the perceived availability of resources for dealing with those demands; therefore, total parenting stress is explained by both parent and child characteristics and situational variables. Feeling overwhelmed, feeling unconfident in the parenting role, and feeling unsatisfied with one’s relationship with a difficult child can all be indicators of parenting stress. With the recent involvement of fathers in the daily care of their children, parenting stress may become a common experience for men, particularly if parenting constitutes a key feature in the development of their full sense of self (Pasley et al., 2002). Moreover, for fathers, increased societal expectations, demands, and responsibilities during the postpartum period create stressors (Kim and Swain, 2007).

In our study, parenting stress was shown to have an effect on maternal postpartum depression starting from the child’s third month, whereas it influences paternal postpartum depression only 6 months after postpartum. We hypothesize that this result reflects how fathers’ engagement with their infants becomes more active across time, as compared to mothers. This outcome shows the importance of following parents longitudinally and the necessity of considering the impact of fathers’ psychological distress in the postnatal period when planning efficacious interventions. Interestingly, we also found that fathers’ depression at 3 months had a direct impact on their level of parenting stress at 6 months, while this did not hold for mothers. Such a result further supports the importance of including both fathers and mothers in early assessments of depression in order to promote child and family well-being.

With respect to partner’s depression, a lack of partner support has been found to strongly predict perinatal depression, both antenatally (Agostini et al., 2015) and postnatally (Milgrom et al., 2008). Conversely, closeness; a lack of a conflictual relationship; shared interests, concerns, and connection with others; partner encouragement to obtain help when needed; and partner agreement regarding infant care may be all considered as protective factors (Dennis and Ross, 2006). In sum, reliable and active support from one’s partner may improve his/her psychological and relational satisfaction and gratification, thus enhancing parenting ability.

We must acknowledge some limitations of this study. First, based on previous empirical and clinical findings, we used an exploratory approach aimed at searching for the most parsimonious explanation of PND. Thus, it is possible that alternative models may provide a better explanation of the data. For instance, we did not include partner’s stress in the model because we could find no studies specifically showing the effect of a partner’s parenting stress on one’s own depression. Nevertheless, we believe that such variable should be included in future studies. The literature has demonstrated the impact of low couple satisfaction on maternal depression, and it is likely that couple adjustment would be associated with parenting stress. Therefore, future research should continue to explore other unidirectional and bidirectional models. Second, because all measures in this study were based on self-reports, and some of them specifically referred to experiences from the last week, we may have captured some transient emotional states that do not necessarily refer to clinical conditions. Third, participation in the study was voluntary; therefore, the recruited sample may not be representative of the community population.

In future, it would be useful to extend the longitudinal perspective to delineate any possible differing trajectories of maternal and paternal mood. Indeed, we have no data related to pregnancy that could shed light on the onset of parental mental health (Ammaniti et al., 2006). However, future results from our wider longitudinal study will include assessments at both nine and 12 months, in addition to the evaluation of couple dyadic adjustment. These data will help us to better understand parental emotional states.

It is thus relevant to improve the early detection of mothers and fathers at risk for perinatal symptomatology in order to provide preventive and efficacious interventions. The link between parenting distress, PND, and anxiety in both parents may increase awareness in clinicians and perinatal staff regarding the relevance of promoting support for parenthood and healthy triadic relationships.

## Author Contributions

LV contributed to prepare the study design, to organize the recruitment of the sample, and to write all sections of the manuscript. LR contributed to organize the recruitment of the sample, and to write the manuscript’s introduction, discussion, and references sections. FA contributed to organize the recruitment of the sample, and to write the introduction and discussion sections of the manuscript. CS contributed to prepare the study design, prepared data set, performed statistical analyses, prepared tables and figures, and contributed to write the method and results sections. VF, SM, and EN contributed to the recruitment of the sample and to data collection. LP contributed to organize the recruitment of the sample, and to write the manuscript’s discussion section. FO, AT, and CP contributed to the recruitment of the sample and to data collection. PB contributed to prepare the study design and supervised the research team. LL contributed to prepare the study design, to organize the recruitment of the sample, supervised data collection and the research team. FM, ES, and RT contributed to prepare the study design and supervised the research team. All authors reviewed and approved manuscript for publication

## Conflict of Interest Statement

The authors declare that the research was conducted in the absence of any commercial or financial relationships that could be construed as a potential conflict of interest.
